# Microstructure and Mechanical Properties of an Al–Mg–Si–Zr Alloy Processed by L-PBF and Subsequent Heat Treatments

**DOI:** 10.3390/ma15155089

**Published:** 2022-07-22

**Authors:** Wonseok Yang, Young-Gil Jung, Taeyang Kwak, Shae K. Kim, Hyunkyu Lim, Do-Hyang Kim

**Affiliations:** 1 Korea Institute of Industrial Technology, Incheon 21999, Korea; sonicyg@kitech.re.kr (W.Y.); jyg3602@kitech.re.kr (Y.-G.J.); shae@kitech.re.kr (S.K.K.); 2 RUAN TECH, Bucheon 14544, Korea; tykwak1@ruantech.re.kr; 3 Department of Materials Science and Engineering, Yonsei University, Seoul 03722, Korea; dohkim@yonsei.ac.kr

**Keywords:** aluminum alloy, mechanical properties, microstructure, powder bed fusion

## Abstract

The aim of this study was to develop a new Al–Mg–Si–Zr alloy with a high magnesium content to achieve a wide range of mechanical properties using heat treatment and at a lower cost. Additive manufacturing was conducted using a powder bed fusion process with various scan speeds to change the volumetric energy density and establish optimal process conditions. In addition, mechanical properties were evaluated using heat treatment under various conditions. The characterization of the microstructure was conducted by scanning electron microscopy with electron backscatter diffraction and transmission electron microscopy. The mechanical properties were determined by tensile tests. The as-built specimen showed a yield strength of 447.9 ± 3.6 MPa, a tensile strength of 493.4 ± 6.7 MPa, and an elongation of 9.6 ± 1.1%. Moreover, the mechanical properties could be adjusted according to various heat treatment conditions. Specifically, under the HT1 (low-temperature artificial aging) condition, the ultimate tensile strength increased to 503.2 ± 1.1 MPa, and under the HT2 (high-temperature artificial aging) condition, the yield strength increased to 467 ± 1.3 MPa. It was confirmed that the maximum elongation (14.3 ± 0.8%) was exhibited with the HT3 (soft annealing) heat treatment.

## 1. Introduction

Additive manufacturing is a fast-growing process based on high geometric flexibility and accuracy while minimizing material waste in metal parts; it is being applied in various fields, such as the general industry, construction industry, biomedical industry, automobile industry, and aerospace industry [1,2,3,4]. There are various methods for the additive manufacturing process, such as direct energy deposition (DED), powder bed fusion (PBF), metal extrusion (ME), binder jetting, and sheet lamination. Out of these methods, PBF is the most widely used for additive manufacturing [2].

Aluminum is one of the most abundant elements of the earth’s crust, along with oxygen and silicon, and is widely used due to its high thermal conductivity, ductility, and recyclability. In addition, aluminum alloys, with an excellent strength-to-weight ratio and formability from the addition of elements such as magnesium, copper, zinc, and manganese, are widely used throughout industrial fields [5,6]. However, when it comes to additive manufacturing, interest in aluminum alloys has been initially lower than interest in steel, nickel alloys, and titanium alloys due to the dense surface oxide film, high laser reflectance, and low spreadability [6,7,8]. Therefore, the number of aluminum alloys for additive manufacturing was very limited and comprised mostly aluminum casting alloys (e.g., AlSi_7_Mg, AlSi_10_Mg) [2]. Nevertheless, due to the many advantages of aluminum alloys mentioned above, the demand for their additive manufacturing in various fields has gradually increased. As a result, researchers have started working on the development of newly designed alloys suitable for additive manufacturing. Compositional variation in high-strength commercial aluminum alloys such as 2000, 5000, 6000, and 7000 series alloys was originally studied to improve the processability of these alloys while not compromising on mechanical properties [9]. The representative aluminum alloys that have recently been developed for additive manufacturing are SCALMALLOY^®^RP and Addalloy™, which are based on aluminum–magnesium alloys with the addition of scandium or zirconium. It has been reported that the addition of scandium and zirconium elements can improve strength by improving the microstructure and suppressing hot cracking [2]. The magnesium contents in SCALMALLOY^®^RP and Addalloy™ are 4.6 and 3.6 wt.%, respectively. Magnesium is the major element in aluminum alloys, and it can increase strength by solid solution strengthening and work hardening [2]. However, conventional commercial aluminum–magnesium alloys (AA5000 series) limited the magnesium content to less than 5 wt.% due to increased surface oxidation and reduced machinability [10]. For this reason, traditional aluminum–magnesium alloys are not able to take advantage of the weld cracking sensitivity reduction and heat treatment characteristics that occur when the magnesium content exceeds 6 and 7%, respectively [11,12,13].

On the other hand, silicon, another essential element, is mainly used in aluminum casting alloys because its fluidity increases when it is added to aluminum. In addition, adding silicon to an aluminum alloy for the additive manufacturing process makes it possible to reduce microcracks by reducing the volume change due to temperature changes [14]. Additionally, when magnesium and silicon are added together, they help to improve the mechanical properties through precipitation hardening. Moreover, the magnesium–silicon precipitates effectively increase the mechanical properties at a relatively low cost compared to expensive additive elements [15]. The current demand for aluminum alloys for additive manufacturing is to design alloys of medium strength with significant elongation, similar to the AA6000 series alloys or high-strength and high-toughness alloys, with better properties than the AA7000 series alloys. In addition, low prices and high productivity are required for the expansion of the use of additive manufacturing parts. For this reason, new alloys are needed to satisfy requirements, such as excellent mechanical properties, low cost, and high productivity. Thus, according to the user’s requirements, the Al–Mg–Si–Zr alloy was designed to have low cost and high strength. To take advantage of the high magnesium content, the magnesium content was increased to 7 wt.%, and zirconium was added to obtain the effect of grain refinement and hot cracking reduction. In addition, silicon was added to further increase its strength and productivity.

The objective of this study was to investigate the effect of heat treatments on the microstructure and mechanical properties of a newly developed Al–Mg–Si–Zr alloy for additive manufacturing. This alloy has a lower silicon content than AlSi_10_Mg alloys and a higher magnesium content than the AA5000 series alloys. Consequently, this compositional change could secure higher productivity due to increased silicon content and improved strength with more enhanced precipitation hardening than solid solution strengthening with the addition of a higher magnesium content. Moreover, it confirmed that excellent mechanical properties can be achieved by subjecting one composition to different heat treatment processes. The correlation between the mechanical properties and the microstructure of the tested alloy was also investigated.

## 2. Experimental Procedure

The Al–Mg–Si–Zr alloy powder, developed by ACTS Technologies (Seoul, Korea), was produced by gas atomization (GA) with the chemical composition of Al-7Mg-2Si-1.2Zr (in wt.%); the powder size range was 20 to 53 μm. The chemical composition of the powder was measured by inductively coupled plasma optical emission spectroscopy (ICP-OES), the powder morphology was observed using scanning electron microscopy (SEM), and a laser-based particle analyzer was employed to measure the powder size.

**Figure 1:**
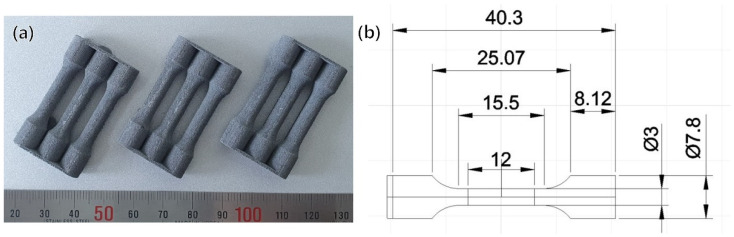
(**a**) As-built specimens, (**b**) geometry of dog-bone specimen (dimension in mm).

To optimize the laser-powder bed fusion (L-PBF) parameters, dog-bone-shaped specimens with a diameter of 4 mm, gauge length of 12 mm, and total length of 42 mm (shown in Figure 1a) were fabricated with different laser scan speeds for mechanical property evaluation and density measurement. The specimens were additively manufactured in an argon gas atmosphere (O_2_ concentration maintained under 0.15%) using a DaegunTech dpert M135 (Changwon, Korea) operating with a laser power of 170 W, a laser beam diameter of 100 μm, a layer thickness of 30 μm, a hatch spacing of 150 μm, a scanning speed from 300 to 475 mm/s, and a volumetric energy density (V_ED_) from 79.5 to 125.9 J∙mm^−3^. For the scanning strategy, a 5 mm chessboard was applied; the rotation between adjacent layers was 67° and the build plate temperature was heated to 150 °C. Moreover, the powder was dried at 200 °C for 2 h before additive manufacturing. Based on the results of this study, it was determined that the optimal scan speed for the Al-7Mg-2Si-1.2Zr alloy was 350 mm/s (V_ED_: 107.9 J∙mm^−3^). This speed is about 100 mm/s faster than the scan speed of the Al–Mg–Sc–Zr alloy without Si in the previous study [16]. Using this optimal scan speed, specimens for the optimization of a post-heat treatment were additively manufactured.

The heat-treatment process was conducted at various temperatures and times to achieve optimal heat treatment conditions for three targets, and all specimens were air-cooled. Each heat treatment condition is listed in Table 1. For the HT1 condition, artificial aging was performed at 180 °C for 4 h to increase the strength with Mg_2_Si precipitation. However, the purpose of the HT1 heat treatment was to minimize the reduction of elongation and increase the strength at the same time. The HT2 process was used to obtain additional Al_3_Zr precipitation. In addition, the target of the HT3 condition, soft annealing, was to attain similar mechanical properties to the AA6000 series alloys.

The microstructures of the as-built specimens under post-heat treatment conditions were observed by electron backscatter diffraction (EBSD) and transmission electron microscopy (TEM). For the EBSD analysis, the specimens were ground and polished following standard techniques. The grain size and orientation were measured using EBSD with a Hitachi SU5000 field-emission scanning electron microscope (FE-SEM). The EBSD scanning was conducted at 500 × 500 μm of the area and 0.3 μm of the step size. Data were analyzed using OIM Analysis (v8; AMETEK EDAX) software information, including the average grain size, which was obtained. The detailed microstructures of the various post-heat treatment conditions were examined using TEM (Talos F200X; FEI) operated at 200 kV with energy-dispersive X-ray spectroscopy (EDS, Super X system; Bruker), and the specimens were prepared using conventional mechanical polishing and ion-milling equipment (Gatan, Model 600).

Dog-bone specimens were machined to evaluate the effect of each heat-treatment condition, as shown in Figure 1b. The machined tensile specimen had a diameter of 3 mm, a gauge length of 12 mm, and a total length of 30 mm. Tensile tests of the additive manufactured specimens were conducted using the universal tensile testing machine at room temperature with an initial strain rate of 10^−3^ s^−1^, and elongation was manually measured for the fractured test specimens. Moreover, three specimens for each condition were tested, and the average values are shown in Figures 3b and 12b.

## 3. Experimental Results

### 3.1. Powder Characterization

**Figure 2:**
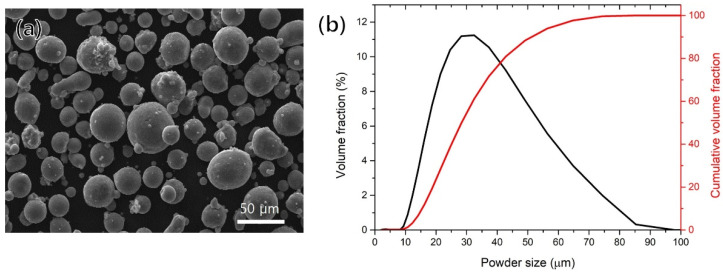
(**a**) SEM image of the powder morphology, (**b**) distribution of particle size.

Table 2 shows the chemical composition of the powder; The measured contents of Mg and Si in the as-built specimen were slightly lower than their contents in the original powder. To confirm the powder size and distribution, an analysis was performed using a laser particle analyzer, and the D_10_, D_50_, and D_90_ of the particles accounted for 19.0, 34.7, and 62.5 μm, respectively, as shown in Figure 2b. Although the powder size distribution was almost within the specified range, some large-sized powder (over 63 μm) was measured, with a volume fraction of about 6%. The SEM images of the powder (Figure 2a) show its morphology; while mostly spherical powders were observed, there were some small amounts of elongated or non-spherical shapes. In addition, some small satellite powders were also observed, which were attached to the powder. These satellite powders were created by vortices near the nozzles when the pressure was excessive during the atomizing process. These non-uniform powders can cause various defects during additive manufacturing but did not significantly affect the powder spreadability in this study.

### 3.2. Optimization of the PBF Process

**Figure 3:**
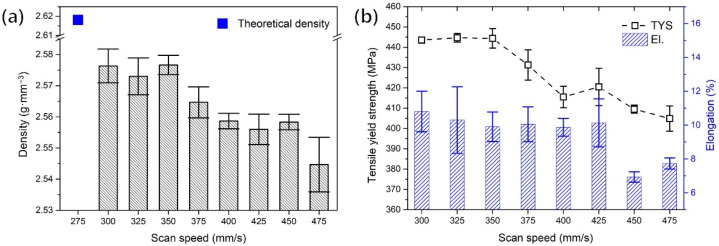
Comparison of various scan speeds: (**a**) relative densities, (**b**) TYS and elongation.

To find the optimal additive manufacturing process, specimens were fabricated at various scan speeds (300 to 475 mm/s), and their properties were evaluated. The density of specimens was measured with the Archimedes method using the electronic densimeter (MD-300, AlfaMirage). The absolute densities were measured by measuring the weight of the samples in the air and water at room temperature. Figure 3a shows the measured densities for the various scan speeds. There was no significant change in density up to a scan speed of 350 mm/s, but above a scan speed of 375 mm/s, the density showed a tendency to decrease with an increasing scan speed. Moreover, as shown in Figure 3b, it was confirmed that the tensile yield strength (TYS) sharply decreased above the scan speed of 375 mm/s. Based on these results, the additive manufacturing conditions of the specimen used to evaluate characteristics of the post-heat treatment process were determined: a laser power of 170 W, a laser beam diameter of 100 μm, a layer thickness of 30 μm, a hatching spacing of 150 μm, and a scanning speed of 350 mm/s (the V_ED_ of this condition was 107.9 J∙mm^−3^).

### 3.3. Microstructure of the as-Built Condition

**Figure 4:**
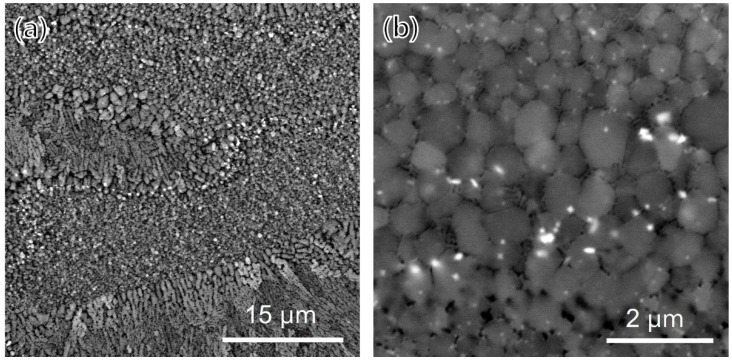
SEM images of (**a**) low magnification and (**b**) the fine-grain region.

Figure 4 shows the microstructure of the as-built specimens. As is well known, the microstructure is divided into fine-grain regions and coarse-grain regions [2,8]. Fine-grain regions (shown in Figure 4b) were located at the bottom of the melt pools and consist of equiaxed grains and two types of sub-micron precipitates. The bright-colored precipitates were observed in the grain boundaries, and dark-colored precipitates with a lamella structure were observed along the grain boundaries. Meanwhile, columnar grains were observed growing along the build direction in the coarse-grain area, and only dark-colored precipitates were observed.

**Figure 5:**
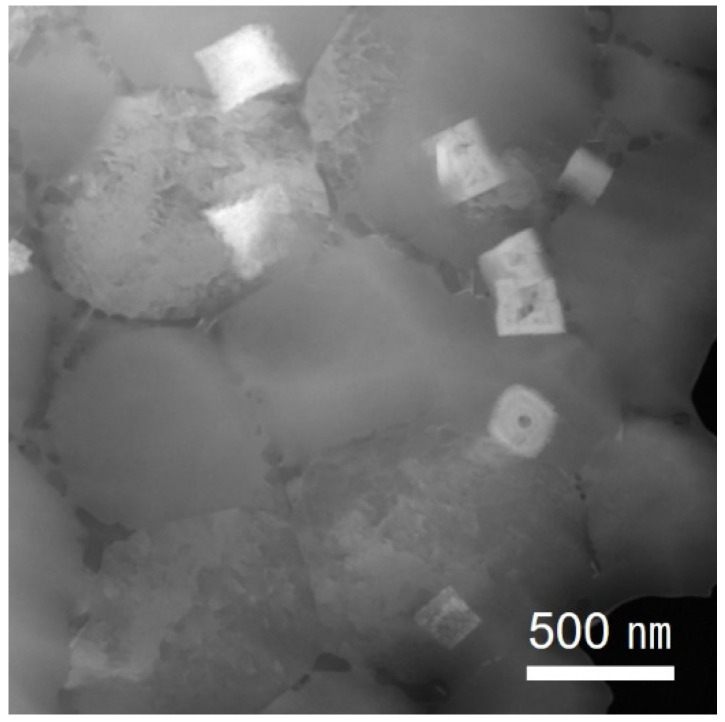
Low-magnification BF-TEM image of the fine-grain region.

**Figure 6:**
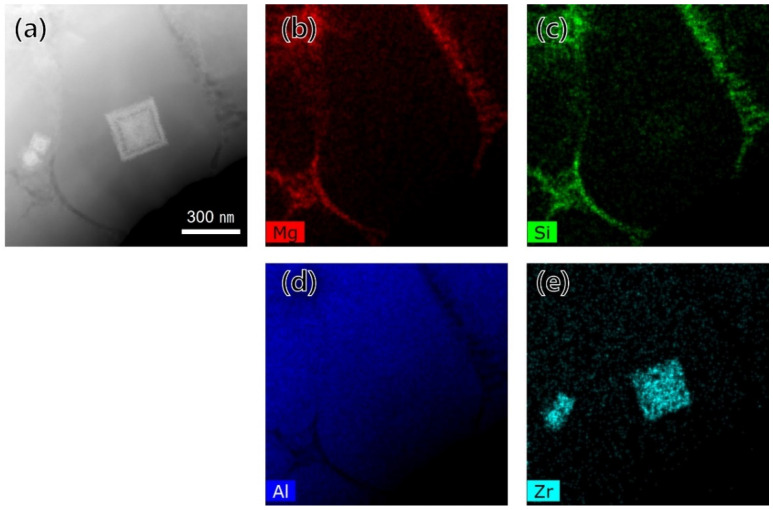
TEM-EDS mapping of a precipitate in the fine-grain region: (**a**) BF-TEM image, (**b**) Mg, (**c**) Si, (**d**) Al, and (**e**) Zr contents.

**Figure 7:**
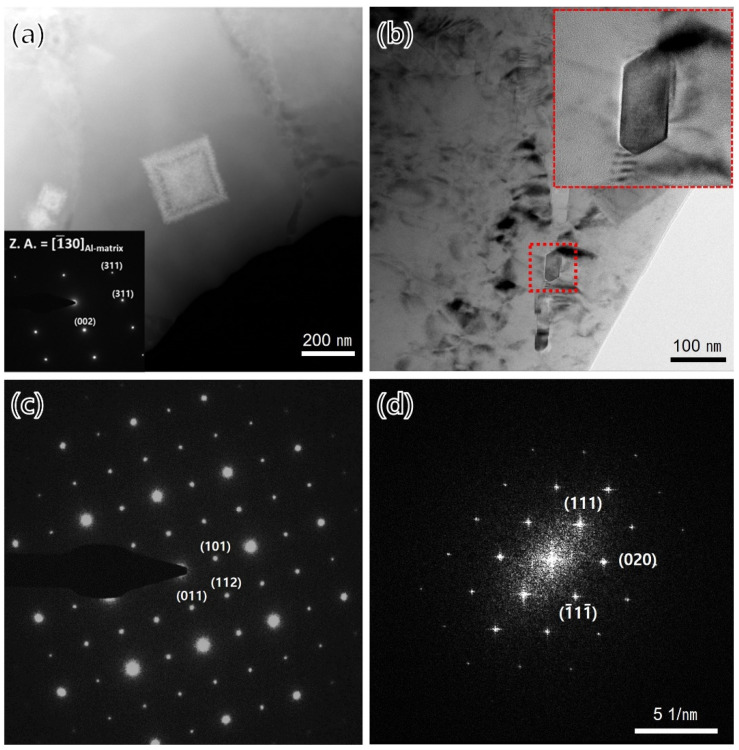
BF-TEM images using the STEM mode and corresponding selected area diffraction (SAD) pattern in the fine-grain region: (**a**,**c**) Al_3_Zr and (**b**,**d**) Mg_2_Si precipitates.

Figure 5 shows a low-magnification bright-field TEM image of the fine-grain region of the as-built specimens. Two types of bright-colored precipitates were observed, and they were divided into a shape in which small spherical precipitates were gathered and there was a large cuboidal shape. The TEM-EDS mapping of the precipitates is shown in Figure 6; the bright-colored cuboidal precipitates were enriched with zirconium, and the dark-colored precipitates were enriched with magnesium and silicon. Figure 7a,b shows the bright-field TEM images of the zirconium-enriched phase and magnesium–silicon-enriched phase. Figure 7c,d shows the corresponding selected area electron diffraction pattern of these phases in Figure 7a,b. The lattice structure of the Al_3_Zr phase was tetragonal with a unit cell parameter of a = 0.3998 nm, and the consequent interplanar spacing of the (101) planes of the Al_3_Zr phase was 0.390 nm. The selected area electron diffraction pattern shown in Figure 7c was observed from the zone axis of [1,2,3,4,5,6,7,8,9,10,11] and indicated a 0.414 nm interplanar spacing of the (101) planes. From this result, it was measured to be about 5.9% larger than the interplanar spacing of the (101) plane on the Al_3_Zr phase. Moreover, the interplanar spacing of the (112) plane was measured to be about 8.0% larger than the theoretical spacing. Nevertheless, it was confirmed that the intermetallic phase shown in Figure 7a was the Al_3_Zr phase. In addition, the lattice structure of Mg_2_Si, another important phase, was face-centered cubic (FCC), with a unit cell parameter of a = 0.6351 nm, and the consequent interplanar spacing of the (−11–1) planes of the Mg_2_Si phase was 0.3675 nm. The selected area diffraction pattern (Figure 7d) was observed from the zone axis of (1–10), indicating a 0.3690 nm interplanar spacing of the (−11–1) plane, which agreed well with the interplanar spacing of the (−11–1) plane of the Mg_2_Si phase.

### 3.4. Microstructure after Post-Heat Treatment

**Figure 8:**
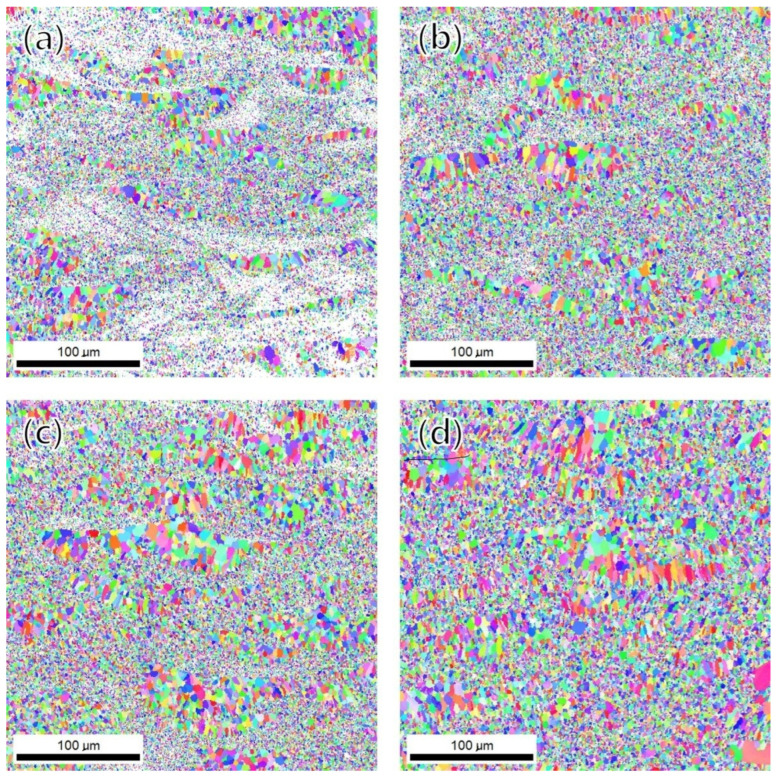
EBSD inverse pole figure (IPF) orientation maps corresponding with grain boundaries on the longitudinal section: (**a**) as-built, (**b**) HT1, (**c**) HT2, and (**d**) HT3 conditions.

**Figure 9:**
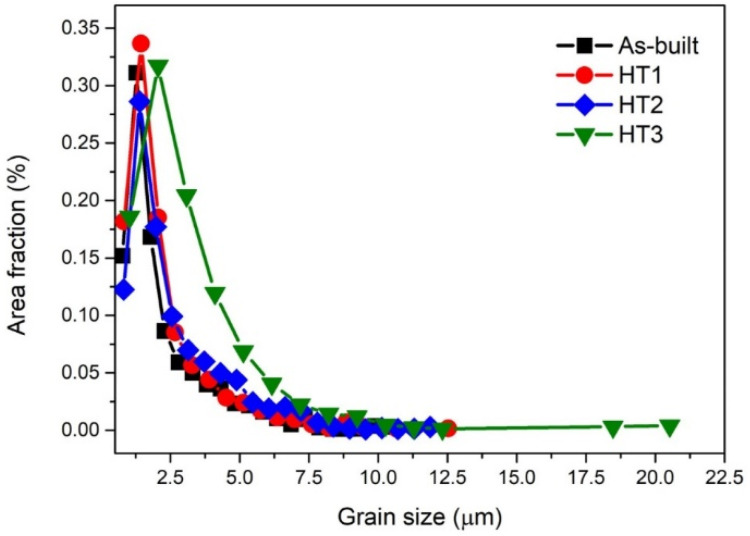
Grain size distribution of the samples under different heat treatment conditions.

The inverse pole figure images of the as-built and post-heat-treated specimens are presented in Figure 8. The average grain size of the as-built, HT1, HT2, and HT3 specimens were 2.242 ± 1.640, 2.266 ± 1.634, 2.601 ± 1.793, and 3.186 ± 2.374 μm, respectively. In the case of low-temperature artificial aging of the precipitate Mg_2_Si phase, the grain size did not change significantly. On the other hand, it was observed that the grain size increased as the heat treatment temperature increased for the Al_3_Zr phase precipitation and soft annealing. As shown in Figure 9, the small change in grain size was mainly due to the growth of fine grains under about 5 μm. However, in the case of the HT3 condition, grain coarsening occurred, as whole grains and coarse grains over 20 μm were also observed. In the low-temperature heat treatment, grain coarsening was suppressed by grain growth pinning of the Mg_2_Si and Al_3_Zr phases located around the grain boundary. However, when the heat treatment was performed at high temperatures, the effect of grain growth pinning was reduced due to the coarsening of the Mg_2_Si and Al_3_Zr phases, resulting in faster grain growth.

**Figure 10:**
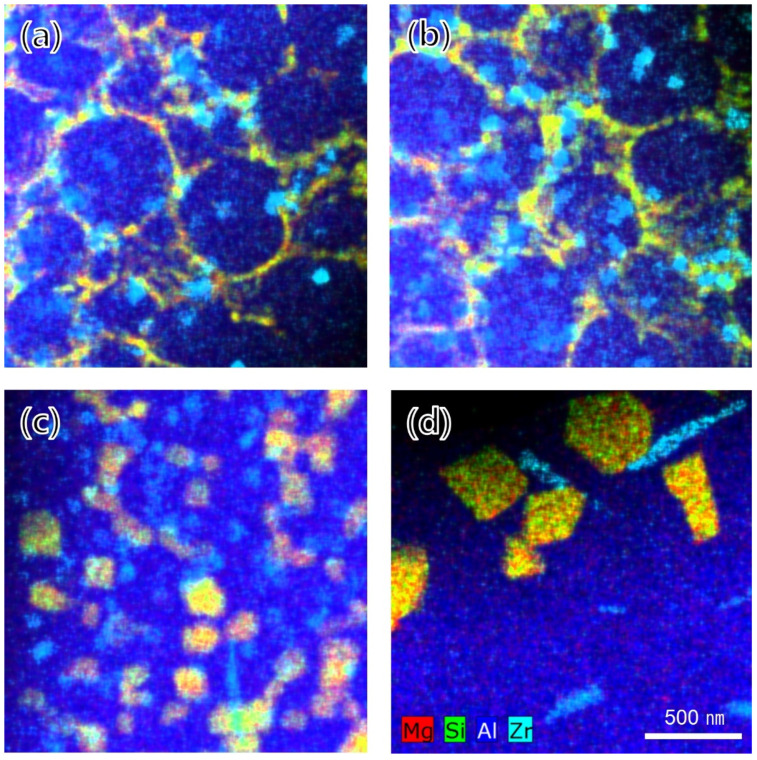
TEM-EDS chemical maps in the fine-grain region: (**a**) as-built, (**b**) HT1, (**c**) HT2, and (**d**) HT3 conditions.

**Figure 11:**
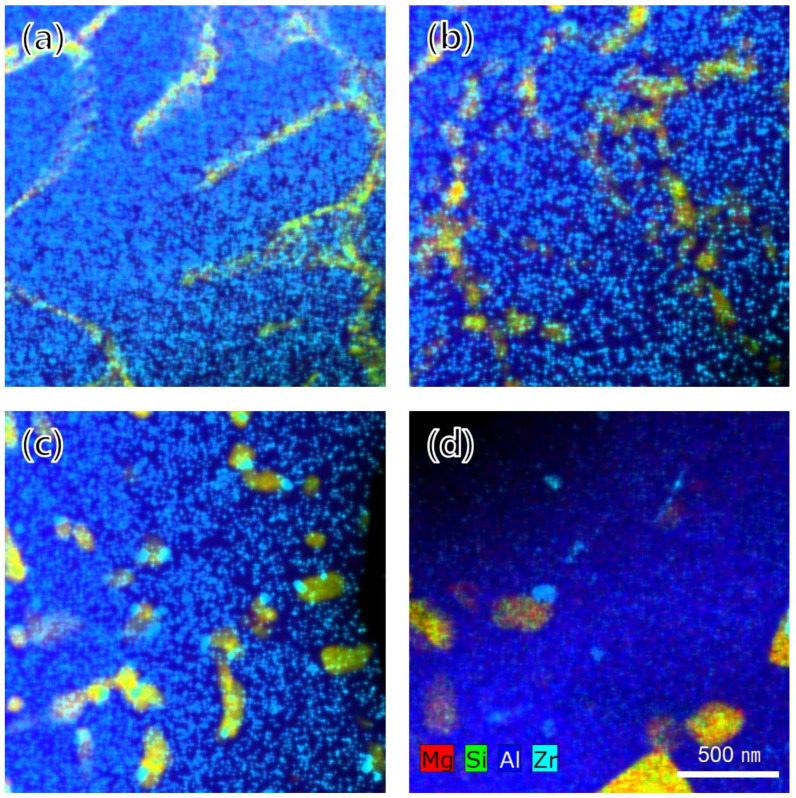
TEM-EDS chemical maps in the coarse-grain region: (**a**) as-built, (**b**) HT1, (**c**) HT2, and (**d**) HT3 conditions.

Figure 10a–d shows the TEM-EDS mapping images of the phases in the fine-grain region. Until the HT2 condition, which was the heat treatment section for precipitation, a large number of Al_3_Zr phases existed uniformly across the entire scanned region regardless of the grain boundaries or aluminum matrix. Meanwhile, Al_3_Zr phases significantly increased in size under the HT3 condition upon subjection to soft annealing. Moreover, under the HT2 condition, it was observed that a morphological change from a cubic to rod-like shape also occurred and that the numbers of rod-like Al_3_Zr phases increased further under the HT3 condition. In addition, it was observed that the Mg_2_Si phase, which was perceived as a continuous eutectic phase along the grain boundaries under the as-built condition, lost continuity of the phase after the HT2 condition and gradually coarsened. The TEM-EDS results for the phase change compared to the coarse-grain region are shown in Figure 11a–d. In contrast to the fine-grain region, Al_3_Zr phase was not observed under both the as-built and HT1 conditions. However, Al_3_Zr phase was observed in the coarse-grain region under the HT2 condition for the Al_3_Zr precipitation heat treatment and slightly coarsened under the HT3 condition with soft annealing.

### 3.5. Mechanical Properties

**Figure 12:**
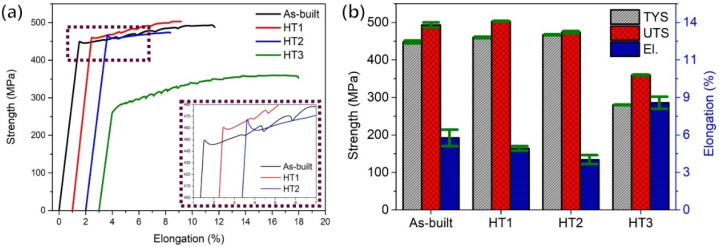
Variation in mechanical properties: (**a**) stress–strain curve and (**b**) average tensile test values.

Figure 12a shows the results of the tensile stress–strain curve to failure in the as-built and various heat treatment conditions for the additively manufactured Al–Mg–Si–Zr alloy. The corresponding average values of the tensile yield strength, ultimate tensile strength (UTS), and elongation at fracture are shown in Table 3. The stress–strain curve of the as-built condition exhibited a clear strain hardening region after the initial elastic deformation until the failure occurred. The average TYS, UTS, and elongation were 447.9 ± 3.6 MPa, 493.4 ± 6.7 MPa, and 9.6 ± 1.1%, respectively. In the case of the HT1 condition, in which Mg_2_Si phases precipitated out, the TYS and UTS increased from 447.9 to 460.0 and 493.4 to 503.2 MPa, respectively. However, the elongation decreased from 9.6 to 8.2%. Meanwhile, in the case of the HT2 condition for Al_3_Zr phase precipitation, the yield strength increased only slightly compared to that of the HT1 condition, but the tensile strength and elongation both decreased. Finally, in the case of the HT3 condition for heat treatment to balance strength and elongation, the yield strength and tensile strength sharply decreased from 460.0 to 280.3 and from 503.2 to 359.8 MPa, respectively, while the elongation increased from 8.2 to 14.3%.

## 4. Discussion

### 4.1. Specimen Density and Defectiveness

As a result of the chemical composition analysis of both the powder and as-built specimens, it was confirmed that the magnesium content of the specimen was 6.17 wt.%, which was reduced by about 8.3% compared to the powder. This reduction in Mg content is related to the well-known fact that magnesium vaporizes during the additive manufacturing process due to the high vaporization pressure of magnesium. A much higher loss of Mg amount was reported by Croteau et al. [17], whereby the amount of magnesium was reduced by about 25% in the PBF process, from 3.6 to 2.7 wt.%. Thus, the loss of magnesium content through vaporization is inevitable in the laser-based additive manufacturing process. Since a decrease in the magnesium content deteriorates the mechanical properties, the reduction in the magnesium content can be suppressed by lowering the V_ED_. However, a low V_ED_ led to the deterioration of the mechanical properties, as it increased the formation of pores in the as-built specimen. As shown in Figure 3a, it is clear that the density of specimens decreased as the scan speed increased. As a result, due to the incomplete melting of the powder layer, pores inside the specimen increase due to the reduction of the V_ED_, and these coarse and irregularly shaped pores reduce mechanical properties [5]. When a load is applied perpendicularly to the long axis of irregular pores, peeling occurs along the long axis of the pores, which sharply reduces the elongation [18]. As a result, as shown from the scan speed of 375 mm/s in Figure 3b, as the scan speed increased, the yield strength sharply decreased, and the elongation also gradually decreased. Moreover, in the case of elongation, it showed a sharp decrease at a speed of 450 mm/s.

### 4.2. Investigation of Microstructure

Zirconium has only been used for the grain refinement of conventional aluminum alloys due to its low solubility. However, the importance of zirconium has increased in additive manufacturing, where it is instantaneously heated to a higher temperature and cooled at a faster rate than the conventional melting method. Zirconium has multiple roles in additive manufactured aluminum alloys, such as grain refinement through heterogeneous nucleation, improving the processability by effectively managing thermal stresses, preventing grain growth during the heat treatment, and improving the mechanical properties [17]. Therefore, various research results on aluminum–zirconium phase have been reported. Among conventional aluminum alloys, which have had the addition of a low zirconium content, the shape of Al_3_Zr has mainly been reported as spherical and rod-shaped [19,20], whereas, in recently additive manufactured alloys, which have had a high content of zirconium added, a cuboidal shape has primarily been reported [2,17]. In this study, the Al_3_Zr phase showed both the shape of the existing aluminum alloy and the aluminum alloy for additive manufacturing, as shown in Figure 5. Octor et al. also reported similar results in their study [21]. According to Octor, four categories of L1_2_ crystal structures were observed after the heat treatment of the rapidly solidified ribbon, which can take various forms depending on the rate of grain boundary movement and the lateral diffusion rate of the grain boundary [21]. Therefore, in this study, the analysis was conducted focusing on the cuboidal shape, which is a characteristic of the alloy for additive manufacturing with the addition of a high amount of zirconium content. As shown in Figure 7c, in the results of the electron diffraction pattern of the Al_3_Zr phase, the interplanar spacing of the (101) plane was approximately 5.9% larger than the theoretical interplanar spacing, and the interplanar spacing of the (112) plane was about 8% larger than that of the theoretical interplanar spacing. This phenomenon might be explained by the fact that the (011) plane of Al_3_Zr coincides with the (002) plane of the Al matrix; the (101) and (112) planes of Al_3_Zr had a few misorientations with the matrix due to lattice strain. Shaokun et al. [22] reported a calculated lattice constant of a = 0.4417 nm for the Al_3_Zr phase and a lattice constant of a = 0.4049 nm for aluminum. The difference between them is about 8%, and the Al_3_Zr particles can act as heterogeneous nucleation cores.

### 4.3. Relationship between Microstructure and Mechanical Properties after Post-Heat Treatment

As shown in Figure 12, it has been confirmed that mechanical properties changed according to the various post-heat treatment conditions, and the characteristics according to the heat treatment conditions can be classified as follows. First, in the case of the low-temperature artificial aging heat treatment for precipitating Mg_2_Si, it can be seen that the yield strength slightly increased. However, as shown in Figure 10a,b, additional precipitation of the Mg_2_Si phase was not observed in the matrix, and no significant difference was found compared to the as-built condition. Meanwhile, this phenomenon can be explained by strain aging. Strain aging is a phenomenon in which the strength increases and the ductility decreases when the specimen is heated at a relatively low temperature after plastic deformation at room temperature [23]. When the specimen is manufactured by the additive manufacturing method, the solidification rate is faster than the conventional melting method due to the rapid cooling rate, so residual stress remains inside; it is presumed that strain aging occurs with a low-temperature heat treatment. Second, in the case of using a heat treatment for precipitating Al_3_Zr, the yield strength increased slightly more than that of the HT1 condition, but the elongation and tensile strength were lower. This phenomenon can be explained by the changes in the microstructure. As shown in Figure 11c, it was confirmed that the Al_3_Zr phase was additionally precipitated in the coarse-grain region that was not previously observed. However, as shown in Figure 10c, a part of the existing Al_3_Zr phase was observed as growing, and it was confirmed that the Mg_2_Si phase was also coarsened and spherical. Through this, it can be seen that the yield point phenomenon increased due to the easy formation of Lüders bands (or the Piobert effect), as the Mg_2_Si phase that previously surrounded the grain boundaries becomes spherical [23]. In addition, the previously observed Portevin-LeChatelier (PLC) effect and work-hardening effects were reduced due to the reduction of dissolved solute atoms in the matrix, the reduction of the dislocation density, and the growth of the secondary phases. The PLC effect is a phenomenon characteristic mainly of Al-Mg alloys, in which solute atoms diffuse at a faster rate than the dislocation rate in the specimen, forming an atmosphere around the dislocation and fixing the dislocation [23]. For this reason, a sawtooth shape occurs in the stress–strain curve and appears in various types depending on the strain rate and temperature [23,24]. In addition, according to Nie et al., the PLC effect was observed in an Al–Cu–Mg alloy to which the zirconium content was added over 0.6 wt.% [25]. The PLC effect is a significant problem in commercial metal alloys because it leads to material instability during processing [26]. This phenomenon has been reported in studies of aluminum–magnesium alloys (work-hardening alloys) [26,27,28]. In addition, the grain size increased by about 16% compared to the as-built condition, and also suppressed a further increase in strength. Finally, it can be seen that the yield strength sharply decreased and the elongation increased after the soft annealing heat treatment. As shown in Figure 10d, it can be seen that the Al_3_Zr phase grew into a rod-like shape, and the Mg_2_Si phase grew coarser. Moreover, as shown in Figure 8d, the grain size under the HT3 condition increased by about 42% compared to that under the as-built condition, confirming that plastic deformation can easily occur due to the smooth movement of dislocations. On the other hand, the PLC effect, which disappeared under the HT3 condition, occurred again. This phenomenon indicates that some Mg_2_Si was decomposed in the process of the heat treatment at a high temperature, and this magnesium was dissolved in the matrix. In addition, it can be considered that the atmosphere in the matrix was changed to a state where the PLC effect could appear at the tested strain rate. However, the precise cause needs to be identified through additional experiments.

## 5. Conclusions

The microstructure and mechanical properties of an additive manufactured Al–Mg–Si–Zr alloy with various heat treatment conditions were investigated. The following conclusions can be drawn from this study:

1. By excluding the addition of expensive alloying elements and optimizing the content of magnesium and silicon, which are the most used solute elements in aluminum alloys, we were able to develop a new alloy that can achieve high strength and high elongation by controlling the heat treatment conditions.
2. The acceptable process parameters for additive manufacturing, considering mechanical properties, are the V_ED_ of this condition, which was 107.9 J∙mm^−3^ (a laser power of 170 W, a laser beam diameter of 100 μm, a layer thickness of 30 μm, a hatching spacing of 150 μm, and a scanning speed of 350 mm/s). However, these results are limited to this study, and the optimum conditions can be changed with variations in other experimental conditions and improved powder quality.
3. The Al_3_Zr phase, as a heterogeneous nucleation site that refines grains and prevents hot tearing during solidification, exhibited a morphological change from a cubic to a rod-like shape under the HT2 condition. In addition, it was confirmed that the Al_3_Zr phase, which was not observed in the coarse-grain region in the as-built state or after the low-temperature heat treatment (HT1), was precipitated after the HT2 heat treatment condition; that is, the high-temperature heat treatment. Therefore, since the Al_3_Zr phase precipitated at a high temperature, the yield strength of the specimen increased compared to the as-built specimen, despite the additional heat treatment at a high temperature of 420 °C.
4. The mechanical properties could be changed to have high strength or high ductility under various heat treatment conditions of additively manufactured specimens. The specimen under the HT1 condition showed a maximum tensile strength of 503.2 ± 1.1 MPa, the specimen under the HT2 condition showed a maximum yield strength of 467.1 ± 1.3 MPa, and the maximum elongation of 14.3 ± 0.8% was achieved under the HT3 condition.

## Figures and Tables

**Table 1:** Summary of post-heat treatment conditions.

Designation	Heat Treatment Condition	Target
HT1	180 °C for 4 h	Precipitation of Mg_2_Si phase
HT2	180 °C for 4 h + 420 °C for 12 h	Precipitation of Al_3_Zr phase
HT3	180 °C for 4 h + 480 °C for 12 h	Soft annealing

**Table 2:** The chemical composition of the powder and as-built specimens.

Elements	Mg	Si	Zr	Al
Powder	6.73	1.96	0.98	Bal.
As-built	6.17	1.83	1.00	Bal.

**Table 3:** Tensile test results of various heat treatment conditions.

Heat Treatment Condition	TYS (MPa)	UTS (MPa)	El. (%)
As-built	447.9 ± 3.6	493.4 ± 6.7	9.6 ± 1.1
HT1	460.0 ± 1.8	503.2 ± 1.1	8.2 ± 0.3
HT2	467.1 ± 1.3	473.9 ± 3.3	6.7 ± 0.6
HT3	280.3 ± 0.8	359.8 ± 1.5	14.3 ± 0.8

## Data Availability

Not applicable.
